# DWI - histology: a possible means of determining degree of liver fibrosis?

**DOI:** 10.18632/oncotarget.24981

**Published:** 2018-04-13

**Authors:** Niklas Verloh, Kirsten Utpatel, Michael Haimerl, Florian Zeman, Claudia Fellner, Marc Dahlke, Philipp Renner, Timo Seyfried, Martina Müller, Christian Stroszczynski, Matthias Evert, Philipp Wiggermann

**Affiliations:** ^1^ Department of Radiology, University Hospital Regensburg, Regensburg, Germany; ^2^ Department of Pathology, University Regensburg, Regensburg, Germany; ^3^ Center for Clinical Trials, University Hospital Regensburg, Regensburg, Germany; ^4^ Department of Surgery, University Hospital Regensburg, Regensburg, Germany; ^5^ Department of Anaesthesia, University Hospital Regensburg, Regensburg, Germany; ^6^ Department of Gastroenterology, University Hospital Regensburg, Regensburg, Germany

**Keywords:** magnetic resonance imaging, liver, abdomen, fibrosis, histology

## Abstract

**Objectives:**

The aim of this study was to determine the diagnostic value of diffusion-weighted MRI of the liver at 3T to classify liver fibrosis/cirrhosis.

**Methods:**

62 patients who underwent both histopathological examination and diffusion-weighted imaging of the liver via 3T MRI within a period of 3 months were included in the study. The Ishak score (1-6) was used to determine the degree of fibrosis: No liver fibrosis (NLF; Ishak 0, n = 16), mild liver fibrosis (MLF; Ishak 1-2, n = 23), advanced liver fibrosis (ALF; Ishak 3-5, n = 12), and liver cirrhosis (LC; Ishak 6, n = 11).

**Results:**

The corresponding ADC values for the individual patient groups were as follows: NLF: 1123 (SD 95.8); MLF: 1032 (SD 77.6); ALF: 962 (SD 68.8); LC: 1015 (SD 60.2) mm2/s. There is a significant difference between NLF and MLF (p = 0.004) and between MLF and ALF (p = 0.022). A significant difference between patients with ALF and LC (p = 0.117) could not be found.

**Conclusion:**

Liver fibrosis/cirrhosis lowers the ADC values of the liver parenchyma in 3T MRI. However, the degree of fibrosis can only be conditionally determined on the basis of ADC values.

## INTRODUCTION

Liver cirrhosis is the irreversible end stage of liver fibrosis. It is characterized by destruction of the lobular and vascular architecture and nodular regeneration of liver tissue. Fibrosis of liver tissue results in extracellular accumulation of collagen fibers, proteoglycans, and other macromolecules [1]. The progression of liver fibrosis and the development of liver cirrhosis are currently viewed as a dynamic process: With adequate treatment of the primary disease, partial remission of liver fibrosis can even be achieved [2–4]. Tests showing restriction of the liver function early, allow for an adjustment of patient treatment, resulting in slowing or regression of the dynamic fibrosis process.

Liver biopsy is currently the gold standard in clinical practice for detecting and staging liver fibrosis/cirrhosis. However, liver biopsy, as an invasive method, has poor patient acceptance, is susceptible to sampling errors, is subject to interobserver variability, and bears a risk of complications, such as infection and bleeding [5]. The absence of characteristic fibrotic septae and nodular configurations can complicate the histological diagnosis of liver fibrosis/cirrhosis [6]. Moreover, sampling errors can underestimate the severity of the disease [7, 8].

With regard to the image-based diagnosis of fibrosis, abdominal ultrasound should be mentioned in particular. Ultrasound elastography (US-RTE) can be used to measure liver stiffness, thus allowing an indirect assessment of the degree of liver fibrosis [9, 10]. However, the diagnostic value of US-RTE is restricted here by the limited reproducibility and the examiner dependence of the method [11].

In addition to ultrasound imaging, MRI of the liver currently represents the gold standard of diagnostic methods. Recently published studies showed that liver fibrosis/cirrhosis can be characterized by liver MRI using a hepatospecific MR contrast agent [12–15].

Individual studies examined the possibility of using diffusion-weighted MRI examination at 1.5T for the classification of fibrosis [16–19]. We are currently not aware of any study examining the correlation of DWI and ADC values on a 3-Tesla MRI unit with histology.

## RESULTS

In total, 62 patients were included in this retrospective study. 16 patients did not show any fibrotic remodeling of the liver parenchyma. 46 patients had a liver parenchyma with fibrotic/cirrhotic remodeling. Table 1 shows the distribution of patients according to Ishak score with the corresponding ADC values.

**Table 1:** ADC values categorized according to Ishak group

Ishak		N	Description	Mean ADC ± SD
**0**	**NLF**	16	No fibrosis	1123 ± 95.8
**1**	**MLF**	12	Fibrosis expansion of some portal areas ± short fibrous septa	1020 ± 77.8
**2**		7	Fibrosis expansion of most portal areas ± short fibrous septa	1024 ± 52.2
**3**	**ALF**	4	Fibrosis expansion of most portal areas with occasional portal to portal (P-P) bridging	1085 ± 109.6
**4**		7	Fibrosis expansion of portal areas with marked portal to portal (P-P) bridging as well as portal to central (P-C)	959 ± 68.7
**5**		5	Marked bridging (P-P and/or P-C) with occasional nodules (incomplete cirrhosis)	965 ± 76.9
**6**	**LC**	11	Cirrhosis, probable or definite	1015 ± 60.2

Table 1 shows the corresponding ADC values with their standard deviations categorized on the basis of the individual Ishak values and the defined groups. NLF: no fibrosis, MLF: mild liver fibrosis, ALF: advanced liver fibrosis, LC: liver cirrhosis.

**Figure 1:**
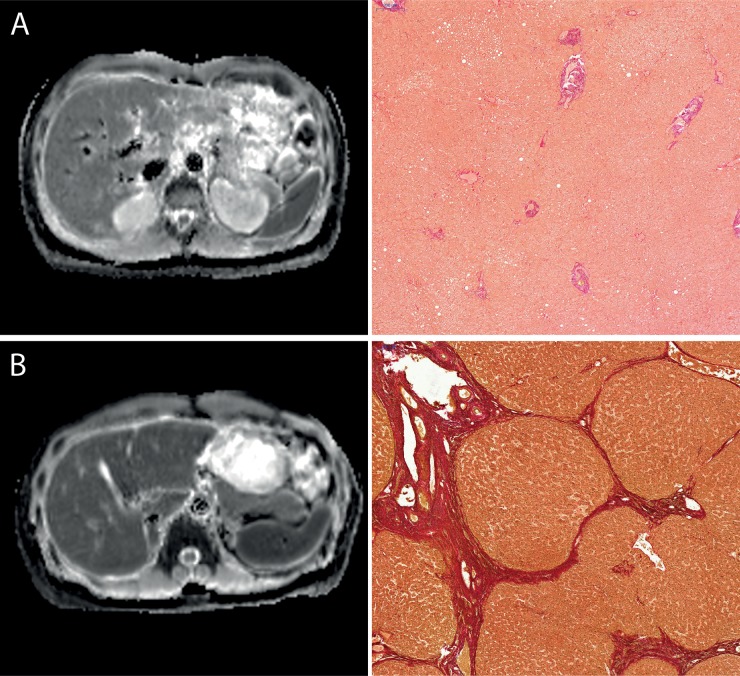
Images comparing the ADC-Maps with corresponding histopathology images with the EVG staining Figure 1 compares the ADC-Maps with corresponding histopathology from a patient with normal liver parenchyma **(A)** and from patient with end stage liver fibrosis **(B)**. All images are with the same window level and center. The ADC-value of the liver parenchyma were as follows: (A) no liver fibrosis (Ishak 0), ADC: 859 mm^2^/s (B) advanced liver fibrosis (Ishak 5), ADC: 1128 mm^2^/s.

There was a significant difference in the mean ADC value between patients without liver fibrosis (ADC = 1123 ± 95.8 mm^2^/s) and patients with any stage of liver fibrosis/cirrhosis (ADC = 1010 ± 76.13 mm^2^/s; p < 0.001) (Figure 1).

**Figure 2:**
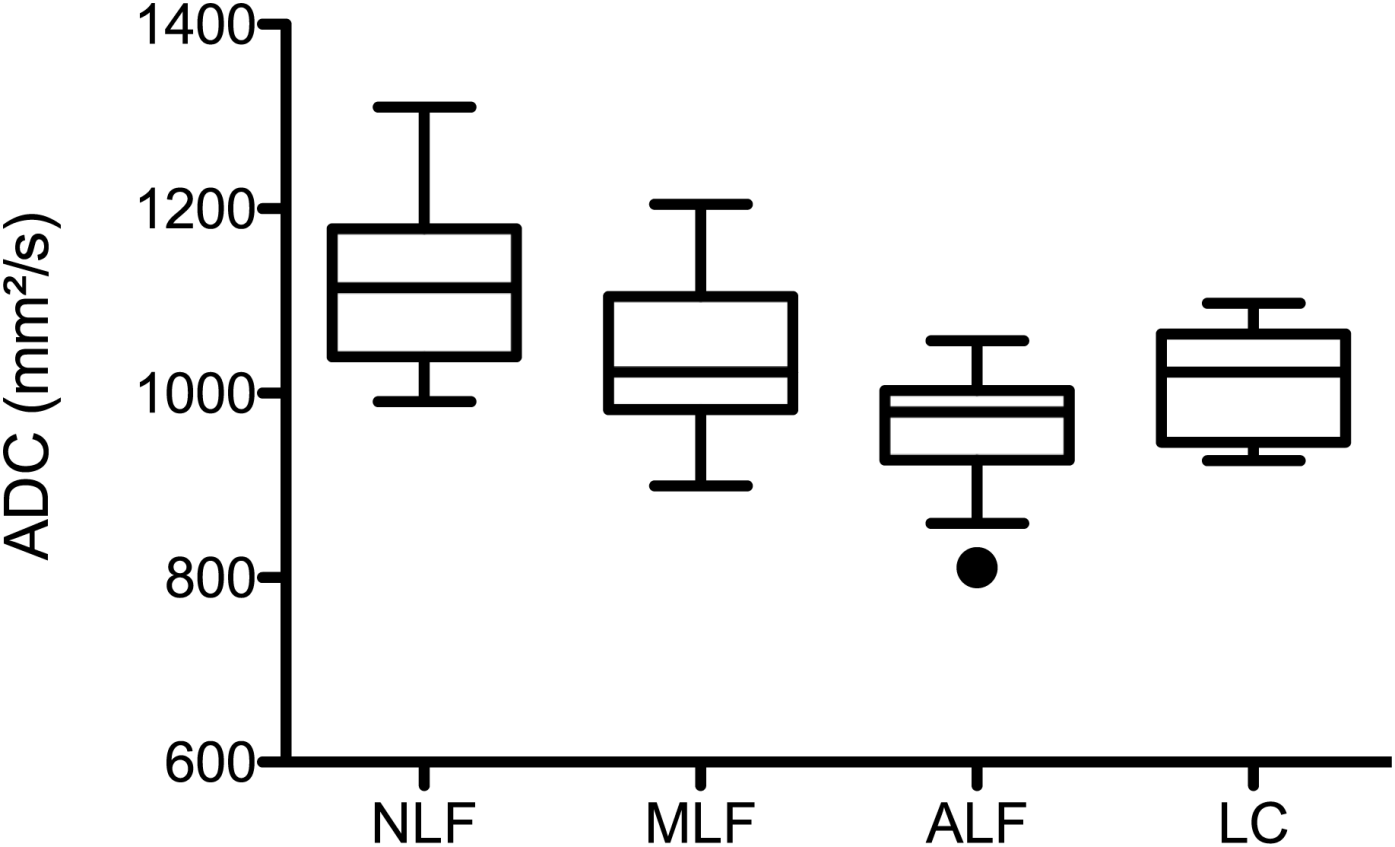
ADC values and liver fibrosis stage Figure 2 shows box plots of the ADC values distributed according to the different fibrosis/cirrhosis groups. The mean ADC ± Standadart diviation values were as follows: no fibrosis, NLF: 1123 ± 95.8; mild liver fibrosis, MLF: 1032 ± 77.6; advanced liver fibrosis, ALF: 962 ± 68.8; liver cirrhosis, LC: 1015 ± 60.2 mm^2^/s.

The corresponding ADC values for the individual patient groups were as follows: NLF: 1123 (SD 95.8); MLF: 1032 (SD 77.6); ALF: 962 (SD 68.8); LC: 1015 (SD 60.2) mm^2^/s (Figure 2). In an adjusted pair-wise comparison (Table 2) a significant difference between patients without liver fibrosis and with mild liver fibrosis (p = 0.036) and between patients with advanced liver fibrosis (p ≤ 0,001) could be observed. No significant difference could be found between the different stages of liver fibrosis/cirrhosis (p > 0.214) or between patients with without liver fibrosis and patients with liver cirrhosis (p = 0.053).

**Table 2:** Adjusted pair-wise comparison

	NLF	MLF	SLF	LC
NLF		**≤ 0.001**	**0.036**	0.053
MLF	**≤ 0.001**		0.214	0.838
SLF	**0.036**	0.214		1.000
LC	0.053	0.838	1.000	

Table 2 shows the corresponding p-values between the defined groups. NLF: no fibrosis, MLF: mild liver fibrosis, ALF: advanced liver fibrosis, LC: liver cirrhosis.

**Figure 3:**
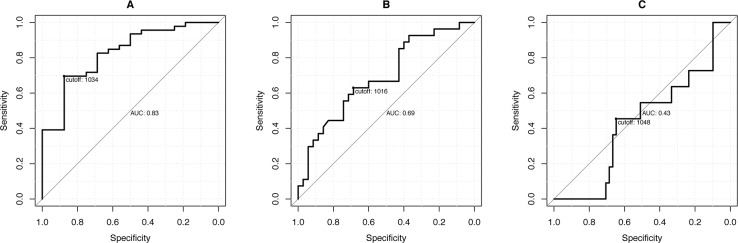
ROC analysis The graphs show the ROC curves with the corresponding sensitivity and specificity levels of the ADC values for the patient diagnoses **(A)** (Ishak ≥ 1; cut-off: 1034), **(B)** (Ishak ≥ 3; cut-off: 1016) and **(C)** (Ishak ≥ 4; cut-off: 1048).

The ROC analysis (Figure 3, Table 3) showed significant cut-off values for the differentiation of Ishak ≥ 1 and Ishak ≥ 3. In the case of an ADC value of 1034, patients without liver fibrosis could be differentiated from patients with liver fibrosis/cirrhosis ≥ Ishak 1 with a sensitivity of 87.5% and a specificity of 69.5%.

**Table 3:** Sensitivity and Specificity of ROC-Analysis

	Ishak ≥ 1	Ishak ≥ 3	Ishak = 6
ADC cut-off	1034	1016	1048
Sensitivity (%)	87.5	68.6	64.7
Specificity (%)	69.6	63.0	45.5
AUC (95% CI)	82.6 (71.0, 94.2)	69.0 (55.7, 82.4)	43.1 (25.2, 61.1)
p-value	p ≤ 0.001	p = 0.002	p = 0.478

Table 3 show the corresponding sensitivity and specificity levels of the ADC values for the patient diagnoses Ishak ≥ 1 (cut-off: 1034), Ishak ≥ 3 (cut-off: 1016) and Ishak ≥ 4 (cut-off: 1048).

## DISCUSSION

The present study was able to show that liver fibrosis affects the mean ADC value of the liver parenchyma. However, it was not possible to differentiate the individual degrees of fibrosis based on the present data.

There is a controversial discussion in the literature regarding the extent to which DWI can be used to differentiate between liver fibrosis and cirrhosis. It has been described that ADC values are significantly lower in patients with liver fibrosis/cirrhosis. Sandrasegaran et al., Taouli et al. and Ayse et al. reported a significant difference in ADC values for different stages of liver fibrosis/cirrhosis [16, 20, 21]. In contrast, studies by Boulanger et al. and Soylu et al. were not able to find a correlation between fibrosis stages and ADC values [17, 22].

As already described, the ADC values of the liver parenchyma were lower in 3T MRI in the case of liver fibrosis/cirrhosis in our study. The individual histological stages of liver fibrosis according to Ishak could not be differentiated from one another on the basis of ADC. However, there was a relevant difference between patients NLF and patients with any stage of liver fibrosis/cirrhosis (MLF + SLF + LC), but this significant difference is mainly effected by the NLF and MLF subgroup, as no significant difference between NLF and LC was observed.

Diffusion-weighted imaging is an MRI method that can also be used for detecting inflammatory/tumoral diseases in the abdomen [23, 24].

Liver fibrosis is characterized by destruction of the lobular and vascular architecture and nodular regeneration of liver tissue. Fibrosis of liver tissue results in extracellular accumulation of collagen fibers, proteoglycans, and other macromolecules [1]. In case of early stage fibrosis, Lee at al. reported that liver parenchyma is often edematous cased by new vessels have leaky interendothelial junctions, resulting in an increased portion of proteins and red blood cells in the extravascular space [25]. This process results in an increased deposition of hepatic water content, hypercellularity and increased ratio of free bound water in the liver parenchyma.

DWI measures the diffusion of water molecules in biological tissues and quantifies the water diffusion processes with the diffusion quotients ADC [26–28]. Theoretically, extracellular collagen fibers, glycosamine, and proteoglycans could inhibit the molecular diffusion of water and thus result in lower diffusion in case of liver fibrosis, especially in early stages [26, 28–30]. In case of liver cirrhosis, these effects might not be as dominant as in case of early-stage liver fibrosis, explaining the lack of significance for patients without liver fibrosis and patients with liver cirrhosis.

The loss of statistical significance regarding the individual degrees of fibrosis based on ADC at 3T is due to the inherent lowering of ADC values at 3T compared to 1.5T. Therefore, the working group of Rosenkrantz et al. described a shortened ADC time for the liver at 3T compared to 1.5T [31]. The influence of the shortened ADC time may explain the fact that the difference between the individual stages of fibrosis in a 3T system is too small for significant differentiation.

Our study has several limitations. First, this study has only a small study population so that the different influencing factors may not have been able to be fully discovered. Second, histological data from both liver biopsies and resections was used. As a result of differences in material quality, there could theoretically be discrepancies in the histological diagnosis. In particular, it can be difficult to differentiate between the individual degrees of liver fibrosis on the basis of liver biopsies.

In conclusion, the degree of liver fibrosis affects DWI particularly in early stage of liver fibrosis, but the corresponding degree of liver fibrosis cannot be determined based on the ADC value.

## MATERIALS AND METHODS

### Patients

In total, 62 patients were included in this retrospective analysis. All patients underwent MR imaging of the liver, either for the purpose of diagnosing suspicious liver lesions or as part of the monitoring of known liver cirrhosis. None of the recruited patients had a contraindication for MRI examination (e.g. claustrophobia, ferromagnetic foreign material, or pacemaker). Only patients who had undergone histopathological examination of the liver parenchyma in a period of 3 months were included in our study.

### Imaging

All examinations were performed on a clinical 3-Tesla system (Magnetom Skyra, Siemens Healthcare). A combination of body and spine coils (18-channel coil, body coil, 24-channel spine coil) was used for signal detection.

To calculate the ADC maps, breath-triggered spin echo EPI sequences for three B-values (50, 400, 800 s/mm^2^) were used with adaptation to body weight using an S (small) and an L (large) program. (S: TR: 5700ms; TE: 52ms; voxel size reconstructed: 1.98 × 1.98 × 6mm; voxel size measured: 2.38 × 1.98 × 6mm; L: TR: 5900ms; TE: 52ms; voxel size reconstructed: 2.08 × 1.08 × 6mm; voxel size measured: 2.51 × 2.08 × 6mm; parallel imaging with an acceleration factor PAT2; averaging performed twice).

### Sequence analysis

To calculate the average ADC time, three circular regions of interest (ROIs) were positioned in the liver. Special attention was paid to prohibit visible vessels, liver lesions, or regions with artifacts. The size of the ROI varied between 1 and 3.5 cm^2^. The thus measured average ADC was viewed as representative for the entire liver.

### Histopathological analyses

Liver biopsies (27), as well as liver resections (35), were included in this study. Liver biopsies were performed as part of active patient monitoring in the case of known liver cirrhosis or an unclear liver tumor. Histological confirmation via surgery was obtained as part of either a liver transplantation or metastasis/liver tumor resection.

All tissue samples were fixed in neutral buffered formalin and embedded in paraffin. Histological slides with a thickness of 4 μm were prepared, deparaffinized with ethanol and xylene, and then stained according to the standard protocols for HE and EVG (Elastica van Gieson). The EVG staining was used to evaluate liver fibrosis. Collagen fibers turn red, while hepatocytes turn yellow.

Needle biopsy was used for the liver biopsy. The length of each biopsy specimen was measured and the number of portal areas was counted. Only liver biopsies with a tissue length of at least 15 mm and 10 portal areas were used. Non-tumorous liver biopsies were exclusively included in the study. Two pathologists specialized in hepatopathology analyzed the liver specimens with respect to the degree of fibrosis. Both examiners were blinded to the radiological data and patient data. Their evaluations were performed independently. In the case of disagreement, scoring was performed again in consensus. The degree of liver fibrosis was determined according to the Ishak scoring system [1].

### Statistical analysis

Statistical analysis was performed using IBM SPSS Statistics (Version 23, Chicago, IL). All data are specified as average ± standard deviation if not otherwise specified. A simple linear regression analysis was performed to correlate the degree of fibrosis/cirrhosis with the DWI data. For the pairwise comparison, the patients were divided into groups on the basis of histological data. To compare the DWI data of subgroups, an Analysis of Variance (ANOVA) followed by Bonferroni adjusted pairwise comparisons was calculated. All statistical analyses were two-sided and a p-value < 0.05 indicated a significant result.

### Key points

Liver fibrosis/cirrhosis affects DWI at 3T.

With increasing liver fibrosis the ADC-Value is lowered.

ADC-Values can partly determined the degree of fibrosis.

## Abbreviations

US-RTE: Ultrasound elastography
MRI: Magnetic resonace imaging
DWI: Diffusion-weighted-imaging
ADC: Apparent diffusion coefficient
ROI: Region of interest
EVG: Elastica van Gieson
NLF: No fibrosis
MLF: Mild liver fibrosis
ALF: Advanced liver fibrosis
LC: Liver cirrhosis
